# REGULATOR: a database of metazoan transcription factors and maternal factors for developmental studies

**DOI:** 10.1186/s12859-015-0552-x

**Published:** 2015-04-10

**Authors:** Kai Wang, Hiroki Nishida

**Affiliations:** Department of Biological Sciences, Graduate School of Science, Osaka University, Toyonaka, Osaka 560-0043 Japan

**Keywords:** Transcription factors, Maternal factors, Development, Database

## Abstract

**Background:**

Genes encoding transcription factors that constitute gene-regulatory networks and maternal factors accumulating in egg cytoplasm are two classes of essential genes that play crucial roles in developmental processes. Transcription factors control the expression of their downstream target genes by interacting with cis-regulatory elements. Maternal factors initiate embryonic developmental programs by regulating the expression of zygotic genes and various other events during early embryogenesis.

**Results:**

This article documents the transcription factors of 77 metazoan species as well as human and mouse maternal factors. We improved the previous method using a statistical approach adding Gene Ontology information to Pfam based identification of transcription factors. This method detects previously un-discovered transcription factors. The novel features of this database are: (1) It includes both transcription factors and maternal factors, although the number of species, in which maternal factors are listed, is limited at the moment. (2) Ontological representation at the cell, tissue, organ, and system levels has been specially designed to facilitate development studies. This is the unique feature in our database and is not available in other transcription factor databases.

**Conclusions:**

A user-friendly web interface, REGULATOR (http://www.bioinformatics.org/regulator/), which can help researchers to efficiently identify, validate, and visualize the data analyzed in this study, are provided. Using this web interface, users can browse, search, and download detailed information on species of interest, genes, transcription factor families, or developmental ontology terms.

**Electronic supplementary material:**

The online version of this article (doi:10.1186/s12859-015-0552-x) contains supplementary material, which is available to authorized users.

## Background

Transcription factors (TFs) bind to the cis-regulatory elements of downstream target genes and promote or block the recruitment of RNA polymerase II to those promoter regions [1,2]. They control various developmental processes by regulating cell fate specification [3,4], morphogenesis [5,6], the cell cycle [7], apoptosis [8] and pathogenesis [9]. Similarly, maternal factors (MFs) present in unfertilized eggs are of interest, as they play crucial roles in early embryogenesis [10-14]. MFs initiate embryonic developmental programs, followed by triggering of zygotic gene activation [10,15,16]. Comprehensive annotation and comparison of TFs and MFs among metazoans would lead to a clearer understanding of developmental processes.

To date, several TF databases, such as AnimalTFDB [17], DBD [18] and TFCat [19], have been established. On the basis of DNA-binding domains (DBD) and sequence similarity, many TFs have been discovered in animals [17], plants [20-23], bacteria [18] and archaea [24]. However, prediction of TFs based only on DNA-binding domains can be misleading, since some non-TF proteins may also have similar domains. For example, the C2H2 type zinc finger domain may also be present in some RNA-binding proteins [25]. Likewise, the homology-based BLAST search method may fail to list every TF in a genome due to the fact that the sequences of some TFs are not so conserved. Therefore, more intelligent methods are needed in order to facilitate better prediction.

The supervised machine learning method combined with feature selection has been demonstrated to be a powerful tool for resolution of various biological problems, especially for placing genes into distinct categories [26,27]. Given that TFs have features such as Pfam ID [28] and Gene Ontology term ID [29] usage that distinguish them from other genes, we have improved the previous method by assigning a different weight to each feature, depending on the category. For example, the GO term GO:0006355 (regulation of transcription, DNA-templated) should appear more frequently in TFs other than non-TFs. This method is based on statistical information similarity (SIS), and its performance has been evaluated.

To gain a better understanding of the roles of every TF and MF, we have developed a developmental ontology browser using the present data, allowing retrieval of information at the cell, tissue, organ, and system levels in a hierarchical way. All developmental ontology terms, as well as other detailed information, can be accessed via the REGULATOR web interface.

## Methods

### Prediction methods for transcription factors

#### Prediction strategy

**Figure 1:**
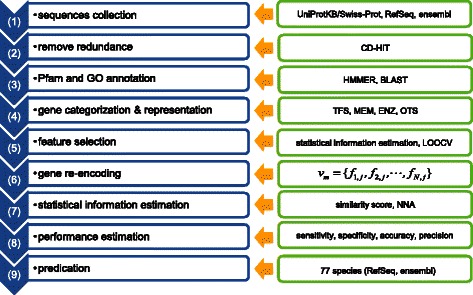
Outline of TF prediction strategy.

The TF prediction workflow employed in the present study using the supervised machine learning method combined with feature selection is shown in Figure 1. First, genes of 77 metazoan species from public databases were collected and redundant sequences were removed. Second, Pfam and GO annotation of the non-redundant sequences were assigned in order to ensure that every protein was represented by at least one feature (Pfam or GO ID). Subsequently, all proteins were categorized into four groups (transcription factors, transmembrane proteins, enzymes, and other proteins), and features that are well represented in each group were selected using feature selection. Third, the weights of annotated features were calculated from the occurrence possibilities for each category. Fourth, every protein was re-encoded according to the selected features. Fifth, TFs based on statistical information similarity were predicted and the performance was evaluated using Leave-One-Out Cross-Validation (LOOCV) [27,30,31] in order to determine features showing the best LOOCV performance. Finally, TFs were predicted using the selected features. Details of these steps are described in the following sections.

### Dataset and preprocessing

Protein sequences of all metazoan genes were collected from the UniProtKB/Swiss-Prot (Release 2013/08), NCBI RefSeq (Release 60) and Ensembl (Release 72) databases. In addition, TF sequences from Ensembl annotated by the Animal Transcription Factor DataBase (AnimalTFDB) [17] were collected as a complement. Amino acid sequences whose length was not between 50 and 5000 or those containing irregular characters (e.g. '*') were excluded. Sequences with high similarity were clustered by CD-HIT [32] at a sequence identity threshold of 0.90. Redundant sequences in each cluster were removed, and only the longest one was retained. These genes were categorized into four groups (transcription factors, transmembrane proteins, enzymes, and other proteins) as the training dataset using the methods described below.

### Pfam and GO annotation

All sequences were searched against the Pfam profile HMM database (Release 27.0) by hmmscan in the HMMER package (v3.1b1) with an e-value threshold of 1e-3. Generally, GO terms could be inferred using either InterProScan or BLAST-based methods. Considering that InterProScan is also based on conserved domains, which are redundant to some degree, we conducted a BLAST-based homology search for GO terms annotation, which provide information complementary to the Pfam domain-based method. All non-redundant proteins were queried against UniProtKB/Swiss-Prot metazoan proteins with BLASTP. Because the number of experimentally validated GO terms is very limited, we also adopted IEAs (Inferred from Electronic Annotation). However, IEAs are often error prone. To ensure more reliable annotation, we used following criteria: (1) We used an e-value of 1e-10 as a threshold. (2) We retained only the top 10 hits. (3) Only GO terms that occurred in no less than 50% of the hit genes were considered to be features of the query gene. (4) Features presented in less than 20 genes were removed. (5) Genes without any features were excluded from the initial training dataset. All of these criteria contribute to support the accuracy. Thus, when inadequate terms were assigned, they would be removed by these criteria. Furthermore, even when minority of GO terms were not correctly assigned, the final score will be determined largely depending on major correctly assigned terms with high weights during the final step of prediction of TFs.

### Classification of genes

In order to clarify the features that distinguish TFs from other proteins, we first categorized the proteins into four groups: transcription factors (TFS), transmembrane proteins (MEM), enzymes (ENZ) and other proteins (OTS) not belonging to any of the first three groups (Table 1). TFS Group: Well-known TFS, including general transcription factors, such as TFIIA, TFIIB, TFIID, TFIIE, TFIIF, and TFIIH [33], were collected from AnimalTFDB (Ensembl IDs), NCBI and UniProtKB/Swiss-Prot based on their functional descriptions or annotations. Then, all Ensembl, NCBI RefSeq and UniProtKB/Swiss-Prot genes whose Pfam or GO descriptions were related to "transcription factor activity" (Additional file 1), or whose names contained the key words "transcription factor" or "transcription initiation factor" were considered to be TFS. Transcription cofactors whose descriptions contained "cofactor", "coregulator", "coactivator" or "corepressor" were categorized into OTS. MEM Group: Proteins whose UniProtKB/Swiss-Prot or NCBI RefSeq descriptions contained "membrane", whose GO terms included "integral to membrane", and whose keywords contained "transmembrane", and those predicted to be transmembrane proteins by TMHMM [34], were considered to be MEM. ENZ Group: NCBI enzymes were identified from the RefSeq descriptions, and UniProtKB/Swiss-Prot enzymes were easily identified from the 'EC' identifier. OTS Group: Homologs (with at least two hits, and no less than half of the top ten hits belonging to at least one category with a BLASTP identity ≥ 25% and an E-value ≤ 1e-20) of the above categories were grouped as TFS, MEM and ENZ, and the other proteins were considered to be OTS.

**Table 1:** **Categories and sample numbers of selected proteins in the training dataset**

**Groups**	**Description**	**Number (Total: 556,753)**
TFS	Transcription factors	64,596
ENZ	Enzymes	119,669
MEM	Transmembrane proteins	269,080
OTS	None of the above proteins	113,892

Note: Some proteins are categorized into more than one group.

### Mathematical representation of genes characterized by features

We have shown a concrete example of mathematical procedures using specific genes in Additional file 2 in order to help understanding what we did in this and following sections. In order to facilitate interpretation by the computational program, a binary gene coding system [27] were employed. Given that a total of N features (a feature being the Pfam or GO term ID) were annotated in a total of M genes, and the features were sorted in alphabetical order, each gene sample was converted to an N dimensional vector, as shown in formulae (1) to (4):

1 $$ {v}_m=\left\{{f}_1,{f}_2,\dots, {f}_i,\dots, {f}_N\right\} $$

2 $$ i\in \left\{1,2,3\dots, \mathrm{N}\right\} $$

3 $$ m\in \left\{1,2,3,\dots, \mathrm{M}\right\} $$

4 $$ {f}_i\in \left\{0,1\right\} $$

where v_m_ is the m-th gene sample out of the total of M samples, and f_i_ is the i-th annotated feature out of the total of N annotated features. If sample v_m_ is annotated with the i-th feature, then f_i_ = 1, otherwise f_i_ = 0.

### Estimation of statistical information

As the frequency of occurrence of each feature differs in each of the four categories (TFS, MEM, ENZ, OTS), the weights of the feature in each category would also differ accordingly. In this study, we measured the weights based on Information content (IC), which has been widely adopted in bioinformatics as well as many other sciences that employ information measuring [35]. Here, statistical information was estimated using the formulae (5) to (9):

5 $$ {P}_{i,j}=\frac{C_{i,j}}{N_j}\cdot \frac{C_{i,j}}{C_1}=\frac{C_{i,j}^2}{N_j\cdot {C}_i} $$

6 $$ I{C}_{i,j}=-{ \log}_2{P}_{i,j} $$

7 $$ {w}_{i,j}=\left\{\begin{array}{cc}\hfill \frac{1}{I{C}_{i,j}},\hfill & \hfill {P}_{i,j}>0\hfill \\ {}\hfill 0,\hfill & \hfill {P}_{i,j}=0\hfill \end{array}\right. $$

8 $$ {W}_{m,j}=\left\{{w}_{1,j},{w}_{2,j},\dots {w}_{N,j}\right\} $$

9 $$ j\in \left\{TFS,MEM,ENZ,OTS\right\} $$

where C_i,j_ is the present frequency of the i-th feature in category j, N_j_ is the total number of sample proteins in category j, and C_i_ is the total number of the i-th feature in the four categories. P_i,j_ is the joint probability of the i-th feature in category j, and it balances both inter-category and intra-category probabilities. IC_i,j_ and w_i,j_ are the information content and weight of the i-th feature in category j, respectively. W_m,j_ is the N dimensional weight vector of the m-th sample in category j. For each sample protein, four weight vectors were assigned because there were four categories and the possibility of each feature being present in each category would differ.

### Feature selection

Next, we tried to select the best features that would yield the best prediction performance. However, feature selection software packages, such as TOOLDIAG [36], mRMR (maximum relevance minimum redundancy) [37] and Weka [38], were time-consuming and incapable of processing large datasets due to the limited memory of our computational server. Therefore, a locally developed Perl pipeline was introduced to carry out this selection. For each feature, we defined MWD_i_ to measure the degree of mutual weight difference between the four categories, as described in formula (10):

10 $$ MW{D}_i={w}_{i,j1}-\left({w}_{i,j2}+{w}_{i,j3}+{w}_{i,j4}\right)/3 $$

where w_i,j1_, w_i,j2_, w_i,j3_ and w_i,j4_ were the sorted weights in descending order of the i-th feature in categories j1, j2, j3 and j4, respectively. Finally, according to MWD_i_, a list of sorted features was generated. In order to reduce the search space, features whose first weight was less than the sum of the others were removed.

Next, LOOCV was carried out and the top best features corresponding to the highest accuracy were selected. Details of this method have been described previously [26,27].

### Prediction based on similarity score estimation

Prediction was carried out using the training data set by estimating and comparing the feature similarity between two proteins. The cosine correlation coefficient function [27,39] was introduced to quantify the similarity of two feature vectors, and a final similarity score was calculated between protein a and protein b, as shown in formulae (11) and (12):

11 $$ si{m}_{\left(a,b\right)}=\frac{V_a\cdot {V}_b}{||{V}_a||\cdot ||{V}_b||} $$

12 $$ SCOR{E}_{\left(a,b,j\right)}=si{m}_{\left(a,b\right)}\cdot {\displaystyle \sum_k^N{w}_{k,j}} $$

where v_a_ and v_b_ represent the N dimensional binary vector of gene a and gene b, respectively, and ||v_a_|| and ||v_b_|| represent the module of vector v_a_ and v_b_, respectively. v_a_ · v_b_ is the product of vector v_a_ and v_b_, and ||v_a_|| · ||v_b_|| is the product of their modules ||v_a_|| and ||v_b_||. k is the k-th feature present in both protein a and protein b. w_k,j_ is the weight of the k-th feature in category j (assuming that protein a is the query, and protein b belongs to category j). Since the weight of each feature differs in each of the four categories, four different scores are obtained. LOOCV was carried out by employing the Nearest Neighbor Algorithm (NNA) classifier [27,39] using the similarity score mentioned above. Query genes were considered to belong to the category with the maximum score.

### Performance evaluation

To evaluate the performance of our predictions, sensitivity, specificity, accuracy, precision and the Matthews correlation coefficient (MCC) [27,40-42] were introduced in this study, as shown in formulae (13) to (17) respectively:

13 $$ sensitivity\kern0.5em =\frac{TP}{TP+FN} $$

14 $$ specifity=\frac{TN}{TN+FP} $$

15 $$ accuracy=\frac{TP+TN}{TP+TN+FP+FN} $$

16 $$ precision\kern0.5em =\frac{TP}{TP+FP} $$

17 $$ MCC=\frac{TP\cdot TN-FP\cdot FN}{\sqrt{\left(TP+FP\right)\cdot \left(TP+FN\right)\cdot \left(TN+FP\right)\cdot \left(TN+FN\right)}} $$

where TP (true positive) is the number of proteins correctly predicted to be TF, FP (false positive) is the number of proteins incorrectly predicted to be TF, TN (true negative) is the number of proteins correctly predicted to be non-TF, and FN (false negative) is the number of proteins incorrectly predicted to be non-TF. The quality was measured by MCC.

We determined features that showed the best LOOCV performance. Finally, TFs were predicted using the selected features in the same way as described above in "Prediction Based on Similarity Score Estimation".

### Prediction methods for maternal factors

To predict MFs, raw data of various normal cell types, tissues and development stages were used. Relevant gene expression series in the Affymetrix Human Genome U133 Plus 2.0 Array (GPL570) and Affymetrix Mouse Genome 430 2.0 Array (GPL1261) were collected from the NCBI Gene Expression Omnibus (GEO) [43]. Background correction and normalization were conducted by GCRMA using the adjusted Robust Multi-array Average (RMA) algorithm [44]. Genes whose expression values were no less than four-fold in unfertilized egg/metaphase II oocytes compared with all late-stage somatic cells were considered to be MFs in order to list egg-specific transcripts, namely strictly maternal transcripts. Late-stage somatic cells excluded embryos at the 1 ~ 8-cell stage, morula stage, blastocyst stage, testis, ovary and embryonic stem cells.

## Results

### TF prediction

**Figure 2:**
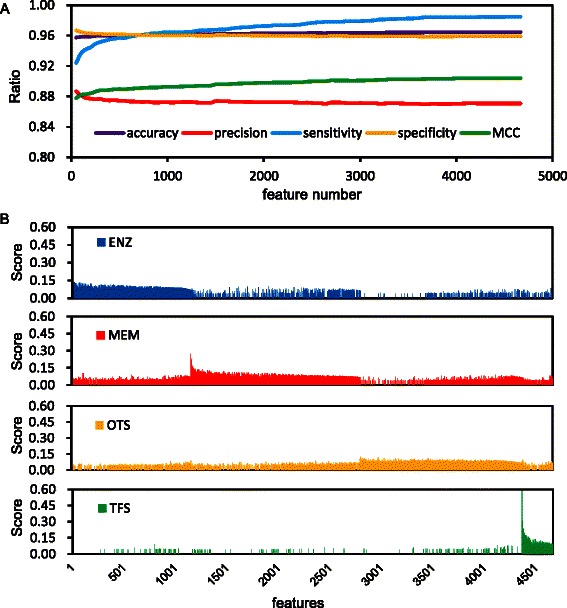
Selected features and performance curves. **(A)** Performance of prediction in the LOOCV. **(B)** Clustering of the 4,666 features according to the similarity score and categories. Blue color indicates the score in ENZ, red color indicates the score in MEM, yellow indicates the score in OTS and green color indicates the score in TFS.

The categories and sample numbers of reserved proteins (total 556,753) in the training dataset are listed in Table 1. These samples were used for subsequent feature selection. As illustrated in Figure 2A, when all of the 4,666 features were selected (Additional file 3), LOOCV accuracy and precision reached 96.5% and 87.1%, respectively, the sensitivity being almost saturated, and the specificity showing no rapid decrease. Clustering of these 4,666 features showed that each group had significantly distinct features (Figure 2B), especially between TFs and non-TFs, thus supporting the high accuracy of our prediction methods. Final prediction was carried out using the sequences of 77 metazoan species (60 from the Ensembl database and 17 from the NCBI RefSeq database). As a result, a total of 85,561 unique TF genes (protein IDs were converted to NCBI GeneID, and if no NCBI GeneID was available, the Ensembl gene ID was used) were identified based on the 4,666 features, and these are summarized in Table 2.

**Table 2:** **Numbers of transcription factors predicted in 77 metazoan species**

**Class**	**Tax ID**	**Organism**	**TF numbers**	**Total genes**	**Percentage (%)**
Aves	9103	Meleagris gallopavo	809	14,123	5.73
	9031	Gallus gallus	941	15,455	6.09
	59729	Taeniopygia guttata	1,291	17,441	7.40
Sauropsida	13735	Pelodiscus sinensis	1,211	18,170	6.66
Reptilia	28377	Anolis carolinensis	1,588	18,575	8.55
Mammalia	9258	Ornithorhynchus anatinus	1,009	21,669	4.66
	9813	Procavia capensis	1,103	16,057	6.87
	9785	Loxodonta africana	1,231	20,003	6.15
	9371	Echinops telfairi	1,106	16,575	6.67
	9986	Oryctolagus cuniculus	1,141	19,213	5.94
	9978	Ochotona princeps	1,029	16,006	6.43
	10141	Cavia porcellus	1,179	18,641	6.32
	10020	Dipodomys ordii	968	15,798	6.13
	10029	Cricetulus griseus	1,307	60,626	2.16
	10090	Mus musculus	1,678	22,716	7.39
	10116	Rattus norvegicus	1,491	22,401	6.66
	43179	Ictidomys tridecemlineatus	1,236	18,786	6.58
	9544	Macaca mulatta	1,593	21,859	7.29
	9555	Papio anubis	1,585	21,785	7.28
	9595	Gorilla gorilla	1,537	20,873	7.36
	9606	Homo sapiens	1,757	22,030	7.98
	9597	Pan paniscus	1,416	20,476	6.92
	9598	Pan troglodytes	1,498	18,672	8.02
	9601	Pongo abelii	1,505	20,370	7.39
	61853	Nomascus leucogenys	1,451	18,534	7.83
	9483	Callithrix jacchus	1,520	20,935	7.26
	39432	Saimiri boliviensis	1,462	19,344	7.56
	9478	Tarsius syrichta	961	13,628	7.05
	30608	Microcebus murinus	1,128	16,319	6.91
	30611	Otolemur garnettii	1,490	19,447	7.66
	37347	Tupaia belangeri	1,005	15,471	6.50
	9615	Canis familiaris	1,402	19,786	7.09
	9669	Mustela putorius furo	1,342	19,872	6.75
	9646	Ailuropoda melanoleuca	1,360	19,317	7.04
	9685	Felis catus	1,321	19,459	6.79
	9739	Tursiops truncatus	1,266	16,550	7.65
	9913	Bos taurus	1,402	19,900	7.05
	9823	Sus scrofa	1,353	21,390	6.33
	30538	Vicugna pacos	730	11,765	6.20
	132908	Pteropus vampyrus	1,219	16,990	7.17
	59463	Myotis lucifugus	1,248	19,679	6.34
	9365	Erinaceus europaeus	843	14,601	5.77
	42254	Sorex araneus	713	13,187	5.41
	9796	Equus caballus	1,343	20,408	6.58
	9361	Dasypus novemcinctus	988	22,711	4.35
	9358	Choloepus hoffmanni	822	12,393	6.63
	9305	Sarcophilus harrisii	1,354	18,779	7.21
	13616	Monodelphis domestica	1,666	21,299	7.82
	9315	Macropus eugenii	973	15,290	6.36
Amphibia	8364	Xenopus tropicalis	1,241	18,346	6.76
Sarcopterygii	7897	Latimeria chalumnae	1,225	19,562	6.26
Actinopterygii	8090	Oryzias latipes	1,281	19,677	6.51
	8083	Xiphophorus maculatus	1,450	20,375	7.12
	8128	Oreochromis niloticus	1,551	21,420	7.24
	69293	Gasterosteus aculeatus	1,317	20,787	6.34
	31033	Takifugu rubripes	1,359	18,484	7.35
	99883	Tetraodon nigroviridis	1,408	19,602	7.18
	8049	Gadus morhua	1,309	20,095	6.51
	7955	Danio rerio	2,376	26,239	9.06
Petromyzontida	7757	Petromyzon marinus	534	10,415	5.13
Ascidiacea	7719	Ciona intestinalis	485	16,652	2.91
	51511	Ciona savignyi	441	11,616	3.80
Echinoidea	7668	Strongylocentrotus purpuratus	763	21,156	3.61
Enteropneusta	10224	Saccoglossus kowalevskii	526	22,077	2.38
Arachnida	34638	Metaseiulus occidentalis	554	11,451	4.84
Insecta	7070	Tribolium castaneum	519	9,761	5.32
	7227	Drosophila melanogaster	662	13,792	4.80
	7463	Apis florea	488	9,137	5.34
	7460	Apis mellifera	318	10,618	2.99
	30195	Bombus terrestris	529	9,433	5.61
	132113	Bombus impatiens	530	9,859	5.38
	143995	Megachile rotundata	530	9,178	5.77
	7425	Nasonia vitripennis	528	11,450	4.61
	7029	Acyrthosiphon pisum	717	15,611	4.59
Chromadorea	6239	Caenorhabditis elegans	782	20,541	3.81
Hydrozoa	6087	Hydra magnipapillata	441	16,826	2.62
Demospongiae	400682	Amphimedon queenslandica	227	9,768	2.32

### MF prediction

MFs are already present in unfertilized eggs, and become gradually reduced as embryogenesis progresses. It has been estimated that about 60% of animal genes are expressed in unfertilized eggs [45]. In order to reduce the search space for developmentally important MFs, we focused only on strictly maternal factors, which are specifically expressed at the egg stage. Due to the limited amount of public data that have been collected at various developmental stages, only human and mouse microarray data deposited in the NCBI Gene Expression Omnibus (GEO) [43] were available. For genes examined using more than one probe and showing inconsistent expression levels between the probes, if the expression based on one probe satisfied the MF criterion, we still retained this gene, considering that the discrepancy may have been due to the presence of some alternative splicing isoforms. Finally, 542 MFs from human and 156 MFs from mouse were obtained.

### Comprehensive annotation

In order to provide a comprehensive annotation, some basic information was extracted from the UniProtKB/Swiss-Prot database and GenBank, including the gene name, description of the full name, and the gene ID. For each Refseq gene, we use NCBI GeneID as the unique ID, whereas for some Ensembl genes without GeneID, the Ensembl gene ID was used. In addition, cross-references to other public databases, such as Ensembl, NCBI RefSeq, UniProtKB/Swiss-Prot and KEGG were also related. A comprehensive InterPro annotation (including FPrintScan, HMMPfam, HMMSmart, ProfileScan, PatternScan, SuperFamily, SignalPHMM, TMHMM, Gene3D and so on), GO and 3D structure links to PDB were also described. Protein-protein interaction information was linked to STRING [46], MINT [47], IntAct [48] and DIP [49]. Putative orthologs were predicted using the bidirectional BLASTP best hit method with an e-value of ≤ 1e-20. Paralogs were inferred with a BLAST identity of ≥ 70% and an e-value of ≤ 1e-50. Moreover, TF targets were also collected from the Transcriptional Regulatory Element Database (TRED) [50] and Embryonic Stem Cell Atlas from Pluripotency Evidence (ESCAPE) [51]. Gene expression profiling of human and mouse TFs in various normal cell types/tissues and at various developmental stages were generated using the same method as that described for MFs prediction.

### Developmental ontology terms

**Figure 3:**
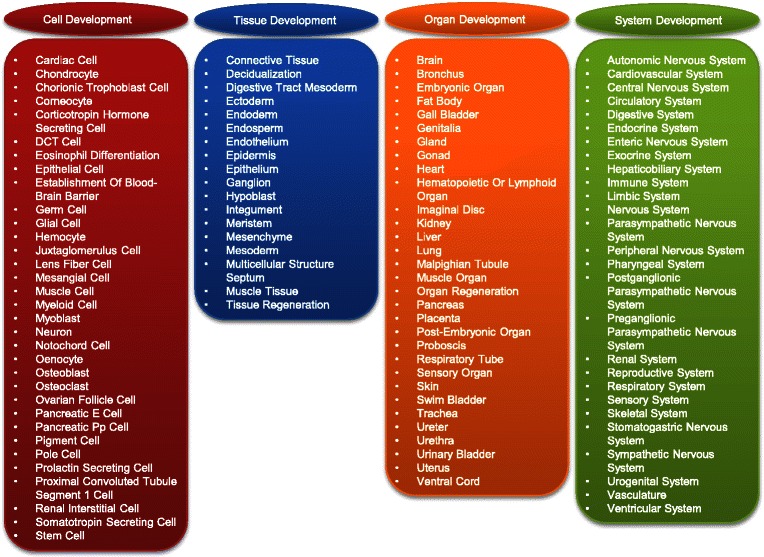
An overview of developmental ontology terms in REGULATOR database. Terms were categorized according to four different development levels: cell, tissue, organ and system.

In order to gain insight into the roles of TFs and MFs during development, the developmental process-associated gene ontology terms were extracted from the Gene Ontology Consortium. These developmental ontology terms would be specifically useful for developmental biology studies. According to their anatomical hierarchies, developmental ontology terms were categorized into four groups: cell, tissue, organ and system (Figure 3). Each of the four groups included many terms other than non-metazoan terms, such as root, leaf and spore germination. Also, all child nodes (e.g. 'is_a' and 'part_of') of the terms were merged.

### Web interface

**Figure 4:**
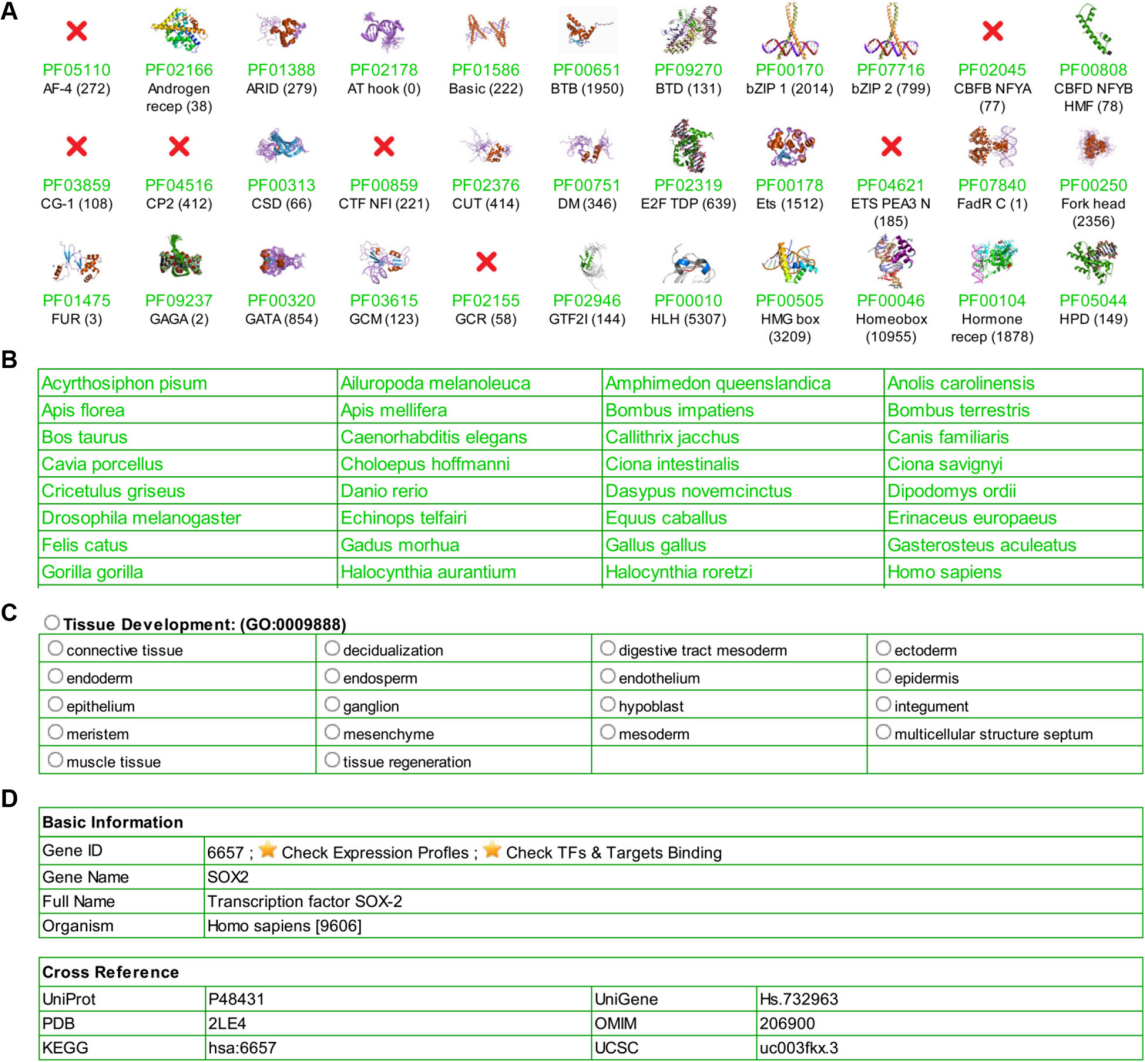
Web interface of REGULATOR. **(A)** Examples of TF families in REGULATOR. **(B)** Available species in REGULATOR. **(C)** Development ontology annotations for Both TFs and MFs. **(D)** Basic information for gene annotations.

To facilitate the use of this resource, a user-friendly web interface (Figure 4) was developed, which can be accessed at http://www.bioinformatics.org/regulator/. By clicking the "Browse" menu, species of all metazoan taxonomic classes used in this study are listed in the left panel. By choosing a species of interest in a certain class, detailed information on the species, including photos, taxonomic classification (kingdom, phylum, class, order, family, genus and species), and the Wikipedia link are shown in the right panel. TFs of all families identified in the species can be accessed via a panel at the bottom. TF families were designated according to the best Pfam DNA-binding domain in the panel. Lists of all TF families are displayed for each species, even when some families are not found in the species, in order to facilitate comparison between species. Using "taxonomic search" at the bottom of the left panel, TFs of selected taxon can be summarized and sorted by their prevalence according to Pfam DBDs (also shown in Additional file 3). Members of each TF family for all available species grouped by the best Pfam DNA-binding domain can also be accessed via the "TF Family" menu. Entire lists of TFs for each species can be accessed via the "Species" menu. MFs of *Homo sapiens* and *Mus musculus* can be accessed via the "Maternal" menu. Expression profiles of the annotated genes in *Homo sapiens* and *Mus musculus* in various tissues and at different developmental stages are also represented in the form of graphs. In addition, ontological representation of every TF and MF was categorized at the cell, tissue, organ, and system levels, and can be searched via the "Ontology" menu. Comprehensive annotations are provided for every TF and MF, including basic information, InterPro, Pfam, Gene ontology annotation, and cross-reference links to many public databases. Users can also search a gene of interest by entering the Gene ID, Ensembl ID, RefSeq ID, gene name, and full name via the "Search" menu. Moreover, InterPro ID, Pfam ID, Gene Ontology ID or key words of their functional annotation are also acceptable. Download and help services and external links to relevant websites are provided.

## Discussion

In this study, we selected the most relevant features that are useful for gene classification from both conserved Pfam domains and sequence similarity-based GO terms. A total of 4666 representative features were obtained, as shown in Additional file 3. As expected, most well-known features of TFs were included among the top 100 features. For example, PF00046 (Homeobox domain), PF00104 (Ligand-binding domain of nuclear hormone receptor), PF00250 (Fork head domain), PF00170 (bZIP transcription factor), GO:0003700 (sequence-specific DNA binding transcription factor activity), and GO:0006355 (regulation of transcription, DNA-dependent) were evident TF features. Furthermore, some other features were also found to be widely present in TFs. For instance, PF01352 (Krüppel associated box) domain-containing proteins were reported as transcriptional repressors in previous studies [52,53]. In addition, reasonable Pfam IDs and GO terms were also found among the top features of other groups (ENZ, MEM, OTS), such as PF00001 (7 transmembrane receptor), GO:0022857 (transmembrane transporter activity) and GO:0004930 (G-protein coupled receptor activity) in the MEM group, and PF07714 (Tyrosine kinase) in the ENZ group. Thus, our statistical information similarity method was capable of distinguishing proteins of different categories.

We then compared our results with other transcription factor databases. Among those whose genome sequences are available, we used 77 metazoan species in the current REGULATOR database, compared with more than 700 species in the DBD database (last updated in 2010) [54] (including eukaryotes, bacteria and archaea) and 50 animal species in the AnimalTFDB (last updated in 2012). Table 3 summarizes the transcription factors of the five model species and compares our prediction with the AnimalTFDB and DBD databases. In human and mouse for example, 1,706 and 1,628 TFs, respectively, were predicted in this study, among which 1,491 and 1,427 TFs were annotated with a previously known Pfam DBD, respectively. The total numbers in REGULATOR are also greater than the 1,494 human and 1,415 mouse TFs in the DBD database [54], and the 1,567 human and 1,507 mouse TFs in the AnimalTFDB database (Ensembl ID being converted to the NCBI GeneID if available). Some genes newly predicted as TFs using our approach might be true TFs. For example, ZBED6 (Zinc finger BED domain-containing protein 6) has been reported to be a transcription factor that can regulate the expression of IGF2 [55,56]. Protein Gm5294 contains a fork-head DNA-binding domain, and may be a transcription factor, although no literature is currently available (57, 58). Similar situations were also found for *Danio rerio, Caenorhabditis elegans* and *Drosophila melanogaster*. We retained these newly predicted genes in our dataset because they share some common features with known TFs.

**Table 3:** **Comparison of transcription factors predicted in this study with those listed in AnimalTFDB and DBD in the five model species**

	**Total**			**Common**			
	**R**	**A**	**D**	**R∩A**	**R∩D**	**A∩D**	**R∩A∩D**
Homo sapiens	1,706	1,567	1,494	1,389	1,097	1,084	1,051
Mus musculus	1,628	1,507	1,415	1,312	1,095	1,093	1,053
Danio rerio	2,376	1,959	1,289	1,564	803	748	688
Caenorhabditis elegans	782	668	736	592	636	582	555
Drosophila melanogaster	662	631	600	513	461	457	425

Note: R: REGULATOR, A: AnimalTFDB, D: DBD.

Further investigation revealed that 111 human and 111 mouse TFs in the AnimalTFDB were not found in our dataset (obsolete gene IDs were not considered). Similarly, 68 human and 77 mouse TFs in the DBD dataset were absent in our data. A manual check of these missing genes revealed that some of them are cofactors or chromatin remodeling factors, rather than true TFs. For example, MBF1 (Endothelial differentiation-related factor 1, ENSMUSP00000015236) in DBD and ATAD2 (ATPase family AAA domain-containing protein 2, ENSG00000156802) in AnimalTFDB were suggested to be transcriptional coactivators in previous studies [57,58]. ZZZ3 (ZZ-type zinc finger-containing protein 3, ENSG00000036549) is a protein of the histone acetyltransferase complex [59], and there is insufficient evidence for it to be a true TF, despite the fact that it is listed in the AnimalTFDB. However, some reliable TFs were still missing from our data, e.g. NFYB, NFYC (Nuclear transcription factor Y subunit beta and gamma). This may have been due to the limited number of features assigned to these proteins. In such cases, we entered them into our database manually. In the sponge, only 227 TFs were predicted (Table 2). The number and proportion of TFs were significantly lower than in other animals. Therefore, the efficiency of our prediction appears to be relatively low for basal metazoans.

We also compared the TFs of *Drosophila melanogaster* in our data with FlyTF database [60] and that of mouse with TFCat database [19], which are curated databases. Among the total 1,168 TFs curated in FlyTF, 581 (50% of FlyTF and 88% of our dataset) were also discovered in our database in which 662 TFs are listed. Manual-check of the 81 TFs only present in our database showed some of them are not TFs. While some others would be genuine TFs [e.g. Tpl94D (geneid:318658) has a HMG-box domain and Aatf (geneid:33943) is an apoptosis antagonizing transcription factor], however, these are not found in the FlyTF. As to the TFs only exist in FlyTF, some of them are TFs [e.g. Mute (FBgn0085444, geneid:2768848) was not predicate by us for lack of predicted TF domain or GO term]. Others may be not TFs [e.g. Blos1 (FBgn0050077, geneid:246439) is a component of biogenesis of lysosome-related organelles complex: Med18 (FBgn0026873, geneid:31140) is coactivator, rather than a TF]. We guess that TFs in the FlyTF database could contain many non-TF proteins because the numbers of Drosophila TFs listed in the AnimalTFDB and DBD are comparable to our data (Table 3). In TFCat, there are 568 mouse TFs that were manually confirmed as reliable TFs. Among them, 429 (76% of TFCat and 26% of our dataset) were commonly shared with our database in which 1,628 TFs are listed. 139 TFs are only exist in TFCat, including both TFs and non-TFs [e.g. Mynf1 (myeloid nuclear factor 1, geneid:104338) is a cell type-restricted transcription factor that is not predicted by us. Trrap (geneid:100683) which belongs to a kinase protein family is not TF. Topors (geneid:106021) is a E3 ubiquitin-protein ligase]. It is likely that TFs in the TFCat database contains only firmly confirmed TFs of limited number, because the numbers of mouse TFs listed in the AnimalTFDB and DBD are relatively similar to our data (Table 3).

The numbers of TFs for each species sorted on the basis of prevalence according to Pfam DBDs are shown in Additional file 4. Among a total of 77 species, 26,300 (31% of total 85,561) in the zf-C2H2 family, 10,955 (13%) in the Homeobox family, 5,307 (6%) in the HLH family, and 3,209 (4%) in the HMG box family were found to be present in our data. This order of prevalence in the top 4 families is well conserved across species.

We also listed MFs specifically expressed in eggs, and provided development ontology annotations. Although many papers have reported the important roles of MFs in various development processes, a large number of MFs are still being investigated and no database has been available to date. In view of their importance and the limited extent of current knowledge, developmental ontology was adopted with the aim of providing a special annotation for these genes. The developmental ontology terms describe developmental processes at four different levels: cell development, tissue development, organ development and system development. All of these terms were extracted from the Gene Ontology consortium [61].

Finally, we have provided a well-annotated database of transcription factors and maternal factors, with cross-database links, functional annotation, protein-protein interactions, gene expression profiles in various tissues and development stages (for human and mouse only).

## Conclusion

In this study, we improved a previous method to detect transcription factors and developed a database include both transcription factors and maternal factors. Ontological representation at the cell, tissue, organ, and system levels has been specially designed to facilitate development studies. This is the original and new in REGULATOR and is not available in other TF databases. We anticipate that these resources will be useful, and will facilitate developmental studies.

## Availability of database

http://www.bioinformatics.org/regulator/

## Additional files

Additional file 1:
**Collected known representative Pfam IDs of transcription factors.** Note: GO:0003700: sequence-specific DNA binding transcription factor activity. GO:0000981: sequence-specific DNA binding RNA polymerase II transcription factor activity. GO:0000982: RNA polymerase II core promoter proximal region sequence-specific DNA binding transcription factor activity.
Additional file 2:
**Examples of mathematical details used in this study.**

Additional file 3:
**Selected representative features for the four categories.**

Additional file 4:
**Numbers of TFs in each species sorted by prevalence according to Pfam DBDs.**

## Abbreviations

TFs: Transcription factors
MFs: Maternal factors
GO: Gene ontology
