# Supplementary material for: REGULATOR: a database of metazoan transcription factors and maternal factors for developmental studies
Source: BMC Bioinformatics. 2015 Apr 10;16:114. doi: 10.1186/s12859-015-0552-x (PMC4411712; doi:10.1186/s12859-015-0552-x)
Supplement: Additional file 2: — Examples of mathematical details used in this study. [file 12859_2015_552_MOESM2_ESM.doc]

**Additional file 2**

**Examples of mathematical details used in this study.**

**Step 1: Mathematical Representation of Genes Characterized by Features**

To show an example, we chose four genes shown below with various annotated Pfam and GO terms:

TFS GROUP:

Sox2:

{PF00505, PF12336, GO:0006355, GO:0043565}

MEM GROUP:

TMEM165:

{PF01169, GO:0006487, GO:0010008}

ENZ GROUP:

NDUFC2:

{GO:0006120, GO:0022904, GO:0044237}

OTS GROUP

GGA2:

{PF02883, GO:0006886}

So the total sorted feature set (including 12 features) is:

{PF00505, PF01169, PF02883, PF12336, GO:0006120, GO:0006355, GO:0006487, GO:0006886, GO:0010008, GO:0022904, GO:0043565, GO:0044237}

If a gene was annotated with the features, the corresponding position in the vector will be 1, otherwise, it will be 0, So,

The vector for Sox2 is: v_1_={1,0,0,1,0,1,0,0,0,0,1,0} corresponding to Formula 1

The vector for TMEM165 is: v_2_={0,1,0,0,0,0,1,0,1,0,0,0}

The vector for NDUFC2 is: v_3_={0,0,0,0,1,0,0,0,0,1,0,1}

The vector for GGA2 is: v_4_={0,0,1,0,0,0,0,1,0,0,0,0}

Thus, structural features (Pfam terms) and annotation features (GO terms) are used for combined inference.

Given that Sox 17 is a test gene, we do not know which group it belongs to.

Sox17 annotation is,

Sox17: {PF00505, GO:0006355, GO:0043565}

The vector for Sox17 is: v={1,0,0,0,0,1,0,0,0,0,1,0} (when a feature was not presented in the above total sorted feature set, just ignore it):

**Step 2: Weight Calculation**

For example, given that there are 2, 4, 6, and 9 protein samples in TFS, MEM, ENZ and OTS category respectively.

For the first feature PF00505,

If PF00505 appears only in one of two protein samples in category TFS, $C_{1,\mathrm{TFS}}=1$

The total number of protein samples in category TFS is 2, so $N_{\mathrm{TFS}}=2$,

The total number of the first feature (PF00505) in the four categories is 1, so $C_{1}=1$,

According to formulae 5-7:

$P_{1,\mathrm{TFS}}=\frac{C_{1,\text{TFS}}}{N_{\text{TFS}}}\cdot\frac{C_{1,\text{TFS}}}{C_{1}}\text{=}\frac{1}{2}\times\frac{1}{1}\text{=}0.5$ Formulae 5

$IC_{1,\mathrm{TFS}}=-\log_{2}P_{1,TFS}=-\log_{2}0.5\text{=}1$ Formulae 6

$w_{1,\mathrm{TFS}}=\frac{1}{IC_{1,TFS}}=\frac{1}{1}=1$ Formulae 7

Similarly,

$$w_{1,\text{MEM}}=0$$

$$w_{1,\text{ENZ}}=0$$

$$w_{1,\text{OTS}}=0$$

And one can also calculate the weights in each category for the 2nd to 12th features. The weight of PF02883 in OTS is, for example, 0.32.

The weight vector of Sox2 are,

In category TFS,

$W_{1,\mathrm{TFS}}=\{1,0,0,1,0,1,0,0,0,0,1,0\}$ Formulae 8

In category MEM,

$$W_{1,\text{MEM}}=\{0,0.5,0,0,0,0,0.5,0,0.5,0,0,0\}$$

In category ENZ,

$$W_{1,\text{ENZ}}=\{0,0,0,0,0.39,0,0,0,0,0.39,0,0.39\}$$

In category OTS,

These weight vector will be used for calculation of the similarity score of Sox2 (Formula 12) in page 4.

**Step 3: Feature Selection and Performance Evaluation**

For the first feature PF00505,

$w_{1,\mathrm{TFS}}=1$, $w_{1,\text{MEM}}=0$, $w_{1,\text{ENZ}}=0$, $w_{1,\text{OTS}}=0$

These are sorted by their weights in descending order.

The mutual weight difference (MWD) in this case is,

$\text{MWD}_{1}=1-(0+0+0)/3=1$ Formulae 10

Similarly,

$$\text{MWD}_{2}=0.5\text{-(0+0+0)/3=0.5}$$

$$\text{MWD}_{3}=0.32\text{-(0+0+0)/3=0.32}$$

……..

……..

$$\text{MWD}_{12}=0.39\text{-(0+0+0)/3=0.39}$$

Features are sorted by their MWD in descending order. {PF00505, PF12336, GO:0006355, GO:0043565, PF01169, GO:0006487, GO:0010008, GO:0006120, GO:0022904, GO:0044237, PF02883, GO:0006886}

For the performance evaluation, features were removed one by one from the end (GO:0006886), and the sensitivity, specificity, accuracy, precision and MCC (Matthews correlation coefficient) are calculated (formulae 13 to 17) each time.

In this study, we evaluated all of the sensitivity, specificity, accuracy, precision and MCC (Figure 2 in the text) and deicide how many features should be used to achieve the best performance. In this study, we used the top 4,666 features.

**Step 4: Prediction Based on Similarity Score Estimation**

We used the cosine similarity (formulae 11) to calculate similarity between the vectors of two genes.

In this case,

Sox17: {1,0,0,0,0,1,0,0,0,0,1,0}

Sox2: {1,0,0,1,0,1,0,0,0,0,1,0}

So,

The cosine similarity of Sox17 to Sox2 is

Fomula 11

$$\text{=}\frac{1\times1+2\times(0\times0)+0\times1+0\times0+1\times1+4\times(0\times0)+1\times1+0\times0}{\sqrt{3\times1^{2}\text{+}9\times0^{2}}\times\sqrt{4\times1^{2}\text{+}8\times0^{2}}}$$

$$\text{=}\frac{3}{\sqrt{3}\times\sqrt{4}}$$

=0.866025404

Note: n is 12 here, because there are 12 features (Pfam IDs and GO IDs) in the total sorted feature set.

Cosine similarity is always between 0 and 1. The cosine similarities of and could be same.

So, we calculate the final similarity score (formulae12) to further distinguish them by introducing weight for the feature in each category (see Supplementary Table S2).

For the three common features of Sox17 and Sox2:

{PF00505, GO:0006355, GO:0043565}

Their weights in four categories are:

TFS: {0.18702612, 0.24017966, 0.21763421}

ENZ: {0.06521342, 0.07171545, 0.00000000}

MEM: {0.04723096, 0.06278823, 0.04456352}

OTS: {0.06319745, 0.09618691, 0.04705002}

Because Sox2 belongs to TFS, we use the weight of TFS.

$score(\mathrm{Sox}17,\mathrm{Sox}2,\mathrm{TFS})=\mathrm{sim}(\mathrm{Sox}17,\mathrm{Sox}2)\cdot\sum_{k}^{N} w_{k,\mathrm{TFS}}$ Formula 12

=0.866025404 x (0.18702612 + 0.24017966 + 0.21763421)

=0.558447813

The vector of:

Sox17: {1,0,0,0,0,1,0,0,0,0,1,0}

TMEM165: {0,1,0,0,0,0,1,0,1,0,0,0}

The similarity of Sox17 to TMEM165 is

=0

Because TMEM165 belongs to MEM, we used the weight of MEM. However, there are no common feature between Sox17 and TMEM165:

$$score(\mathrm{Sox}17,\text{TMEM165},\text{MEM})=\mathrm{sim}(\mathrm{Sox}17,\text{TMEM165})\cdot\sum_{k}^{N} w_{k,\text{MEM}}$$

=0 x (0)=0

The vector of:

Sox17: {1,0,0,0,0,1,0,0,0,0,1,0}

NDUFC2 : {0,0,0,0,1,0,0,0,0,1,0,1}

The similarity of Sox17 to NDUFC2 is

=0

Because NDUFC2 belongs to ENZ, we used the weights of ENZ. However, there are no common feature between Sox17 and NDUFC2:

$$score(\mathrm{Sox}17,\text{NDUFC2},\text{ENZ})=\mathrm{sim}(\mathrm{Sox}17,\text{NDUFC2})\cdot\sum_{k}^{N} w_{k,\text{ENZ}}$$

=0 x (0)=0

The vector of:

Sox17: {1,0,0,0,0,1,0,0,0,0,1,0}

GGA2: {0,0,1,0,0,0,0,1,0,0,0,0}

The similarity of Sox17 to GGA2 is

=0

Because GGA2 belongs to OTS, we used the weights of OTS. However, there are no common feature between Sox17 and GGA2:

$$score(\mathrm{Sox}17,\text{GGA2},\text{OTS})=\mathrm{sim}(\mathrm{Sox}17,\text{GGA2})\cdot\sum_{k}^{N} w_{k,\text{OTS}}$$

=0 x (0)=0

In the case of Sox17, the scores in four categories are,

in TFS group is 0.558447813

in MEM group is 0

in ENZ group is 0

in OTS group is 0

Obviously, 0.558447813 is the largest one, so Sox17 is predicated to be TFS.

In our actual analysis, we calculated the final similarity score between Sox 17 and all the other genes belonging to the four categories, and utilize highest similarity score to decide the category which Sox17 belongs to.
